# Durability of COVID-19 vaccine-induced immunity in Saudi Arabia: evidence, gaps, and implications for Hajj and mass-gathering preparedness

**DOI:** 10.3389/fpubh.2026.1853635

**Published:** 2026-07-22

**Authors:** Leena E. Azhar, Ahdab A. Alsaieedi, Mariam M. AlEissa, Hussain A. Baroom, Amro H. Alghoraibi, Khalid S. Alzahrani, Esam I. Azhar

**Affiliations:** 1 Clinical Sciences Department, MBBS Program, Fakeeh College for Medical Sciences, Fakeeh Care Group, Jeddah, Saudi Arabia; 2 Special Infectious Agents Unit BSL-3, King Fahd Medical Research Center, King Abdulaziz University, Jeddah, Saudi Arabia; 3 Department of Medical Laboratory Sciences, Faculty of Applied Medical Sciences, King Abdulaziz University, Jeddah, Saudi Arabia; 4 Medical School, Department of Medicine, Alfaisal University, Riyadh, Saudi Arabia; 5 Molecular Biology Department, Jeddah Regional Laboratory, Ministry of Health, Jeddah, Saudi Arabia

**Keywords:** cellular immunity, COVID-19 vaccination, Hajj, heterologous boosting, humoral immunity, hybrid immunity, immune durability, mass gatherings medicine

## Abstract

The policy question for COVID-19 vaccination has shifted from whether vaccines induce protection to how long clinically meaningful protection persists and how immune information should guide preparedness. This question is especially relevant to Saudi Arabia, where Hajj and Umrah create predictable periods of dense population mixing among pilgrims with diverse vaccination histories, prior infection exposures, ages, and comorbidity profiles. This structured narrative review synthesizes evidence on durability of COVID-19 vaccine-induced immunity, Saudi epidemiological and serological data, heterologous booster strategies, hybrid immunity, and operational implications for mass-gathering preparedness. A structured search of biomedical databases and public-health sources was undertaken for evidence published from January 2020 to May 2026. The review highlights those circulating antibodies and neutralizing activity decline after vaccination, particularly against antigenically divergent variants, whereas memory B-cell responses and CD4+ and CD8+ T-cell immunity generally provide more durable protection against severe disease. Saudi evidence remains limited but informative: national seroprevalence studies, vaccine-rollout data, clinical-effectiveness analyses, and recent serological work indicate substantial prior exposure, strong post-vaccination antibody responses, and meaningful protection against hospitalization and intensive-care admission, particularly after booster dosing. Heterologous schedules and hybrid immunity appear to broaden immune protection, but their policy relevance lies in flexible risk-based planning rather than in promoting infection as a strategy. For Hajj and Umrah, the main implication is a shift from simple dose counting toward immune durability, risk stratification, booster timing, vaccination documentation, genomic surveillance, and integrated respiratory-virus monitoring.

## 1 Introduction

The global COVID-19 vaccination program began as an emergency effort to prevent severe disease, deaths, and health-system collapse. Early phase 3 trials showed high short-term efficacy of BNT162b2 and mRNA-1273 against symptomatic COVID-19 and established vaccination as a cornerstone of pandemic control (1, 2). As the pandemic progressed, however, SARS-CoV-2 evolved through successive variants and subvariants with variable immune escape. This changed the central question for public-health policy: durable protection, rather than peak immune response alone, became the outcome of greatest practical importance.

Saudi Arabia provides a distinctive setting in which to interpret this question. The national COVID-19 response built on previous experience with Middle East respiratory syndrome coronavirus (MERS-CoV), emergency preparedness, digital health platforms, and mass-gathering medicine (3). By the time this review was revised, open public-health dashboards recorded more than 826,000 confirmed COVID-19 cases and approximately 9,500 reported deaths in Saudi Arabia, while global and national vaccination datasets indicated high vaccine uptake, with more than 180 administered doses per 100 population reported by late 2023 (4–6). These numbers should be interpreted as administrative and surveillance indicators rather than complete measures of infection burden, because testing behavior, reporting systems, case definitions, and denominator estimates changed over the course of the pandemic.

The Saudi context is not only national; it is also international. Hajj and Umrah bring pilgrims from many countries into a setting characterized by travel, crowding, shared accommodation, close contact, physical exertion, and intense movement across ritual sites. These factors can amplify respiratory-virus transmission and create operational pressure on healthcare services (7–10). For the 2025–2026 season, Saudi Hajj health requirements included COVID-19 vaccine or immunity documentation for specified groups and advised Hajj travelers to receive vaccination according to the updated WHO SAGE roadmap (7). This illustrates how COVID-19 vaccination has moved from a crisis-response measure to part of a broader risk-management framework for travel and mass gatherings.

A Saudi-focused review is warranted despite the still-limited volume of Saudi longitudinal immunology data for three reasons. First, Saudi Arabia has generated relevant epidemiological, serological, vaccine-effectiveness, and operational evidence that has not been sufficiently integrated into one preparedness-oriented synthesis (11–17). Second, the Kingdom’s mass-gathering role makes the translation of immune-durability evidence into policy unusually important, even when some mechanistic evidence comes from international cohorts. Third, identifying what Saudi data are missing is itself useful: gaps in longitudinal cellular immunity, variant-specific neutralization, booster durability, and pilgrim-specific studies should guide future national research.

This review therefore has four objectives: to summarize Saudi epidemiological and vaccine-related evidence; to synthesize the coordinated roles of humoral, memory B-cell, and T-cell immunity in durable protection; to interpret heterologous booster and hybrid-immunity evidence for a population with heterogeneous immune histories; and to translate these findings into practical preparedness considerations for Hajj, Umrah, and future respiratory-pathogen threats.

## 2 Methods

### 2.1 Review design

This article was designed as a structured narrative review. A narrative design was appropriate because the evidence relevant to COVID-19 immune durability and mass-gathering preparedness is heterogeneous, spanning clinical trials, immunological cohorts, seroprevalence studies, vaccine-effectiveness analyses, policy reports, and operational guidance. The review did not aim to pool effect estimates. Instead, it sought to interpret evidence across immunology, vaccinology, Saudi public health, and mass-gathering preparedness. The approach was guided by principles for transparent narrative reviews, including a clear objective, explicit search concepts, balanced evidence selection, critical synthesis, and adequate referencing (18).

### 2.2 Search strategy and eligibility

PubMed/MEDLINE, Scopus, Web of Science, and Google Scholar were searched for publications from January 2020 to May 2026. Search concepts included SARS-CoV-2, COVID-19 vaccines, immune durability, waning immunity, neutralizing antibodies, anti-spike IgG, memory B cells, CD4+ T cells, CD8+ T cells, hybrid immunity, heterologous boosting, Saudi Arabia, Hajj, Umrah, mass gatherings, digital health certification, genomic surveillance, hospitalization, intensive-care admission, and mortality. The search was supplemented by authoritative public-health sources, including WHO, the Saudi Ministry of Health, Saudi open-data dashboards, and travel-medicine guidance.

Eligible sources included peer-reviewed clinical trials, cohort studies, observational serological studies, real-world vaccine-effectiveness studies, systematic reviews, meta-analyses, and public-health reports. Priority was given to studies that addressed immune durability, booster responses, hybrid immunity, Saudi epidemiological or vaccine data, and operational implications for Hajj or other mass gatherings. Opinion pieces were used only when they provided policy context or conceptual framing. Studies were excluded when they were unrelated to vaccine-induced immunity, lacked sufficient methodological clarity, or did not contribute to the Saudi or preparedness focus of the review.

### 2.3 Data extraction and synthesis

For each included source, the following information was considered when available: study design, population, country, vaccine platform, prior infection status, booster schedule, sampling interval, immune endpoints, clinical outcomes, and relevance to Saudi Arabia or mass-gathering preparedness. Immune endpoints included binding antibodies, neutralizing antibodies, ACE2-blocking capacity, memory B-cell responses, CD4+ T-cell responses, CD8+ T-cell responses, breakthrough infection, hospitalization, intensive-care admission, and death. The evidence was synthesized across seven integrated domains: the Saudi context; the epidemiological characteristics of Hajj and Umrah; coordinated humoral and cellular immunity; heterologous boosting and hybrid immunity; preparedness translation; limitations; and future research priorities.

## 3 Saudi Arabian epidemiological, vaccination, and immune landscape

Saudi Arabia’s COVID-19 immune landscape was shaped by overlapping waves of infection, rapid vaccine deployment, booster implementation, and later breakthrough infections. The first vaccine used nationally was BNT162b2, approved in December 2020, followed by the Oxford-AstraZeneca ChAdOx1 vaccine in February 2021 (3, 16). The early national plan emphasized phased prioritization, electronic registration, safety monitoring, and scale-up through vaccination centers (3). This operational infrastructure is important because immune durability cannot be interpreted separately from how quickly vaccination was delivered, which platforms were used, and how boosters were timed.

Saudi epidemiological data provide the necessary background for interpreting immune studies. The open Saudi COVID-19 dashboard listed 826,312 confirmed cases, 813,775 recoveries, 3,050 active cases, and 9,487 deaths when accessed during manuscript revision (5). These data do not capture all infections, particularly asymptomatic or untested infections, but they provide a historical picture of the reported epidemic burden. The vaccination rollout achieved high administrative coverage, with open international datasets reporting more than 180 administered doses per 100 population by late 2023 and continuing to track primary-series and booster indicators through WHO-derived sources (6).

Evidence from Saudi seroprevalence studies indicates that infection-derived immunity was already substantial before and during vaccine rollout. A nationwide seroprevalence study reported geographically variable SARS-CoV-2 antibody prevalence, showing that community exposure differed across regions and populations (11). A more recent Saudi serological study evaluating BNT162b2, mRNA-1273, and ChAdOx1 vaccination found baseline SARS-CoV-2 IgG positivity in 75.4% of participants before vaccination, strong post-vaccination antibody responses, and neutralizing antibodies in 98.1% of IgG-positive participants (12). These findings suggest that many real-world Saudi vaccine responses reflected hybrid immune priming rather than vaccine-only exposure.

Clinical-effectiveness data from Saudi Arabia, although not yet as extensive as the international literature, strengthen the local relevance of this review. In a large Saudi cohort, a single dose of BNT162b2 or ChAdOx1 was associated with a high protection rate over 8 months, although the cohort was predominantly young and therefore not fully representative of high-risk pilgrims (13). A retrospective cohort study from Jazan examined real-world effectiveness of two-dose vaccination with BNT162b2 and ChAdOx1 (14). During the Omicron period, vaccination was associated with improved clinical outcomes among infected patients in Saudi Arabia (15), and a nationwide retrospective cohort study showed that booster vaccination enhanced protection against intensive-care admission during the Omicron surge (16). A separate Saudi analysis reported reduced hospital admission among vaccinated COVID-19 patients before Delta predominance (17).

Taken together, Saudi evidence is informative but incomplete. It supports the public-health value of vaccination and boosters, confirms substantial prior exposure, and suggests meaningful protection against severe outcomes. However, Saudi-specific longitudinal data on cellular immunity, variant-specific neutralization, mucosal immunity, booster durability in older adults, and immune responses among international pilgrims remain limited. This limitation is not a reason to avoid a Saudi-focused review; rather, it clarifies why a review is needed to connect existing national evidence with international immunological data and to identify priorities for future Hajj-oriented studies. The Saudi COVID-19 immune landscape should be interpreted as the cumulative result of reported infections, substantial unmeasured community exposure, phased vaccine deployment, booster implementation, and subsequent breakthrough infections. Table 1 summarizes the main national evidence domains that are relevant to immune durability and preparedness planning, including reported epidemic burden, vaccine rollout, seroprevalence, post-vaccination serological responses, and clinical outcomes.

**Table 1:** Saudi COVID-19 evidence relevant to immune durability and preparedness.

Evidence domain	Main observations	Preparedness interpretation
Reported epidemic burden	Open dashboards listed more than 826,000 confirmed cases and approximately 9,500 reported deaths during manuscript revision (5).	Reported cases and deaths provide context but underestimate total exposure because testing and reporting changed over time.
Vaccination rollout	BNT162b2 was introduced in December 2020, followed by ChAdOx1 in February 2021; open datasets indicate high uptake, exceeding 180 administered doses per 100 population by late 2023 (3, 6).	Dose history, platform, and time since last dose should be considered alongside population-level coverage.
Seroprevalence	Saudi seroprevalence studies show substantial and geographically variable prior exposure (11).	Administrative vaccination records alone do not fully represent population immunity.
Post-vaccination serology	A Saudi comparative serological study reported 75.4% baseline IgG positivity, strong post-vaccination responses, and neutralizing antibodies in 98.1% of IgG-positive participants (12).	Saudi immune profiles often represent hybrid exposure and should be interpreted accordingly.
Clinical outcomes	Saudi studies reported vaccine-associated reductions in severe outcomes, hospital admission, and ICU admission, with booster doses strengthening protection during Omicron (13–17).	Preparedness should prioritize severe disease prevention, especially for high-risk residents, pilgrims, and health workers.

This table summarizes Saudi evidence on reported COVID-19 burden, vaccine rollout, seroprevalence, post-vaccination serology, and clinical outcomes. It highlights that population immunity reflects not only vaccine coverage, but also prior infection, booster history, vaccine platform, time since exposure, variants, and host risk factors.

## 4 Hajj and Umrah as epidemiological settings: exposure intensity and risk profile

Hajj and Umrah represent distinct but complementary epidemiological settings for respiratory-pathogen transmission. Hajj is a recurrent international mass gathering involving pilgrims from many countries over a compressed period, whereas Umrah occurs throughout the year and creates repeated opportunities for travel-associated pathogen importation. Both settings include dense crowding, shared transport, shared accommodation, close physical proximity, multilingual communication needs, and frequent movement between ritual locations (8–10).

Respiratory infections are among the most consistent health concerns during pilgrimage. Travel-medicine reviews and CDC guidance note that respiratory tract infections are common during Hajj and that pneumonia is a frequent cause of hospital admission among pilgrims (9, 10). The risk is not driven by crowding alone. It also reflects long-distance travel, sleep disruption, heat exposure, physical exertion, variable baseline health status, and different vaccine access in countries of origin.

The pilgrim population includes groups at higher risk of severe COVID-19 and other respiratory infections, including older adults, pregnant women, persons with diabetes, chronic kidney disease, cardiovascular disease, chronic lung disease, obesity, and immunosuppression. These risk categories are consistent with broader evidence on predictors of severe COVID-19 and with travel-medicine recommendations for respiratory protection in older or comorbid travelers (9, 19, 20). Therefore, Hajj preparedness should not be framed only as prevention of infection transmission. It should also focus on preventing hospitalization, intensive-care admission, death, and disruption of routine healthcare services during predictable periods of increased demand.

This background explains why immune durability matters operationally. If protection against infection wanes faster than protection against severe disease, a mass-gathering strategy that aims to eliminate every infection will be unrealistic. A more appropriate strategy is layered: pre-travel vaccination advice, targeted boosters for high-risk groups, documentation of vaccination or immunity when required, syndromic and laboratory surveillance, genomic monitoring, clear risk communication, and rapid clinical pathways for severe respiratory illness (7–10).

## 5 Durability of vaccine-induced immunity: coordinated humoral, memory B-cell, and T-cell protection

Humoral and cellular immunity are best discussed together because they do not operate as independent compartments. Antibodies help block infection at the point of exposure, memory B cells support rapid secondary antibody responses, CD4+ T cells coordinate B-cell maturation and antiviral immunity, and CD8+ T cells contribute to the clearance of infected cells (21–26). The durability of protection after COVID-19 vaccination is therefore not captured by a single laboratory measurement.

Neutralizing antibodies remain one of the most useful correlates of protection against symptomatic SARS-CoV-2 infection. Khoury and colleagues showed that neutralizing-antibody levels are highly predictive of protection against symptomatic infection (21). Subsequent analyses demonstrated that neutralization levels also help explain reduced protection against immune-escape variants and the benefit of booster doses in restoring protection (22). These findings support the operational value of booster timing when a predictable exposure period is approaching.

Antibody waning, however, should not be equated with vaccine failure. After vaccination, antibody concentrations typically rise, peak, and then decline as short-lived plasmablast responses contract. This is a normal feature of adaptive immunity. What matters clinically is the relationship between antibody decline, variant immune escape, exposure intensity, and host vulnerability. Waning antibodies can increase the probability of breakthrough infection, particularly when a variant is antigenically distant from the vaccine strain, but memory responses may continue to reduce the risk of severe disease (21–26).

Cellular immunity provides an important explanation for the observed difference between protection against infection and protection against severe outcomes. CD4+ T cells support germinal-center responses, affinity maturation, and memory B-cell development. CD8+ T cells recognize and eliminate infected cells, limiting viral replication and tissue injury. T-cell responses target multiple epitopes, many of which are less affected by spike mutations than neutralizing-antibody epitopes (22–25). This broader recognition helps explain why vaccine protection against hospitalization and death has often remained more durable than protection against mild infection, even during variant waves.

Memory B cells add another layer of durability. They may persist and undergo further maturation after infection or vaccination, enabling rapid recall responses upon re-exposure. Longitudinal studies have shown that immune memory to SARS-CoV-2 can be maintained for months after infection and vaccination even when circulating antibody levels decline (23–26). This distinction is central for public communication. Breakthrough infection after vaccination does not mean that vaccination has lost value; it may still convert a potentially severe primary infection into a more controlled secondary immune challenge.

For Saudi Arabia, the policy implication is that serological surveys are useful but incomplete. Anti-spike IgG and neutralization data can help describe population-level humoral immunity, as illustrated by Saudi serological findings (12), but they should be interpreted alongside clinical risk, time since last vaccine or infection, vaccine platform, booster status, variant circulation, hospitalization data, and, when feasible, cellular immune studies. In mass-gathering planning, immune monitoring should be viewed as part of a preparedness intelligence system rather than as a standalone laboratory exercise. Vaccine-induced protection against SARS-CoV-2 is not determined by circulating antibodies alone. Durable protection reflects the coordinated activity of neutralizing antibodies, binding antibodies, memory B cells, CD4+ T cells, CD8+ T cells, and integrated immune memory. Table 2 summarizes the major immune components involved in protection and highlights how each should be interpreted for vaccination policy and preparedness.

**Table 2:** Coordinated immune mechanisms and their relevance to protection.

Immune component	Role in SARS-CoV-2 protection	Policy interpretation
Neutralizing antibodies	Reduce infection and symptomatic disease by blocking viral entry; levels correlate with protection but decline over time (21, 22).	Useful for assessing short-term protection and booster timing, especially before predictable exposure periods.
Binding antibodies	Reflect vaccine or infection exposure and often correlate with neutralizing activity, but do not fully describe functional protection (12, 21).	Helpful for population serology but should not be used alone for individual risk decisions.
Memory B cells	Support rapid antibody recall and may mature over time after antigen exposure (23, 26).	Explain why protection may persist even when serum antibodies decline.
CD4+ T cells	Provide B-cell help, support germinal-centre responses, and coordinate antiviral immunity (24, 25).	Important for durable immune memory and interpretation of vaccine response beyond antibody titres.
CD8+ T cells	Recognize and clear infected cells, contributing to protection against severe disease (24, 25).	Particularly relevant for high-risk groups where preventing severe outcomes is the priority.
Integrated immune memory	Humoral, B-cell, and T-cell responses together determine clinical protection (22–26).	Preparedness should combine immune, clinical, epidemiological, and genomic indicators.

This table summarizes the complementary roles of neutralizing antibodies, binding antibodies, memory B cells, CD4+ T cells, and CD8+ T cells in SARS-CoV-2 protection. It emphasizes that antibody waning does not equal loss of protection, because immune memory contributes to sustained protection against severe disease.

## 6 Heterologous boosters and hybrid immunity: evidence and relevance to pilgrims

### 6.1 Heterologous booster strategies

Heterologous vaccination refers to the use of different vaccine platforms across primary and booster doses. Initially, this approach was often adopted because of supply constraints, safety concerns, or changes in national recommendations. Subsequent evidence showed that heterologous schedules may also provide immunological advantages in some contexts, particularly when an adenoviral-vector vaccine is followed by an mRNA vaccine (27–29).

The Com-COV trial provides key randomized evidence. It was a UK single-blind, randomized, non-inferiority trial that compared homologous and heterologous prime-boost schedules using ChAdOx1 nCoV-19 and BNT162b2 at different intervals. The study showed that heterologous schedules were immunogenic and feasible, although reactogenicity and immune endpoints varied according to vaccine order and interval (27). Its importance for policy lies not only in the antibody results, but also in demonstrating that mixed-platform schedules can be evaluated systematically rather than treated as *ad hoc* compromises.

Pozzetto and colleagues provided complementary real-world and immunological evidence. In a French healthcare-worker cohort of 13,121 participants, heterologous ChAdOx1/BNT162b2 vaccination was associated with better protection against SARS-CoV-2 infection than homologous BNT162b2/BNT162b2 vaccination. The investigators also conducted longitudinal immune analyses showing strong anti-spike and neutralizing responses after the heterologous schedule (28). Because healthcare workers experience high exposure intensity, these findings are relevant to other settings in which exposure is predictable and concentrated.

A 2025 longitudinal study further supports the durability of heterologous boosting. One year after vaccination, heterologous prime-boost regimens were associated with higher anti-spike IgG levels and stronger ACE2-blocking capacity than homologous schedules, and preserved T-cell responses were observed, including stronger CD8+ IFN-gamma responses in heterologous recipients regardless of prior infection status (29).

Heterologous boosting is directly relevant to Hajj and Umrah because pilgrims arrive with diverse vaccine histories. Some may have received mRNA vaccines, others adenoviral-vectored or inactivated vaccines, and many may have incomplete documentation or long intervals since their last dose. A preparedness framework that recognizes evidence-based heterologous boosting can provide flexibility before travel, especially for high-risk pilgrims, healthcare workers, and essential personnel who require rapid strengthening of protection before predictable crowding. Such flexibility must be accompanied by clear communication, pharmacovigilance, documentation standards, and alignment with national and WHO recommendations (7, 27–30).

### 6.2 Hybrid immunity

Hybrid immunity refers to immune protection generated by infection plus vaccination, regardless of sequence. It may arise from infection before vaccination, vaccination followed by breakthrough infection, or repeated antigen exposures over time. Several studies have shown that hybrid immunity can generate broader and more durable humoral and cellular responses than infection or vaccination alone (31–33).

Hybrid immunity is particularly relevant to Saudi interpretation because local serological studies suggest substantial prior exposure before vaccination (11, 12). This means that strong post-vaccination antibody responses in some Saudi cohorts may reflect immune recall after infection rather than vaccine priming in immunologically naive individuals. This observation helps explain why vaccine responses and effectiveness estimates may differ across populations with different epidemic histories.

Hybrid immunity should not be treated as a policy goal. Natural infection carries risks of severe disease, death, long COVID, and healthcare disruption. Its relevance is interpretive: it helps public-health authorities understand why population immunity may be heterogeneous, why simple dose-count categories can be misleading, and why seroprevalence data may improve preparedness modeling. In pilgrimage settings, some individuals may have strong hybrid protection, whereas others may remain vulnerable because of limited vaccine access, advanced age, immunosuppression, comorbidities, long time since last exposure, or exposure to immune-escape variants.

## 7 Translating immune durability into Hajj, Umrah, and respiratory-pathogen preparedness

The practical value of immune-durability evidence lies in how it informs preparedness. For Saudi Arabia, the goal should be a layered system that connects vaccine policy, risk stratification, digital documentation, surveillance, genomic sequencing, clinical readiness, and communication.

First, booster timing should be aligned with predictable exposure periods. Hajj and high-volume Umrah seasons provide clear windows during which respiratory-virus exposure may increase. For high-risk pilgrims, healthcare workers, first responders, and staff working in pilgrimage areas, vaccine assessment should consider not only the number of doses received but also time since last dose or infection, vaccine platform, age, comorbidities, immunosuppression, and circulating variants (7, 9, 19–22, 30).

Second, digital health tools should be used as public-health intelligence platforms, not merely as administrative certificates. Saudi Arabia has substantial experience with digital vaccination documentation and mass-gathering operations. WHO-supported digital health certification initiatives for Hajj pilgrims illustrate the importance of cross-border verification of vaccination and health information (34). When used ethically and securely, digital systems can help identify documentation gaps, support risk stratification, and link vaccination history to surveillance and clinical outcomes.

Third, immune monitoring should be integrated with epidemiological and genomic surveillance. Serological trends can indicate broad changes in population immunity, but they become more useful when interpreted alongside syndromic surveillance, laboratory testing, variant sequencing, hospital admissions, intensive-care trends, and mortality. International systems such as the Global Influenza Surveillance and Response System and GISAID demonstrate the importance of laboratory networks and rapid data sharing for respiratory-virus preparedness (35, 36).

Fourth, COVID-19 should be embedded within integrated respiratory-pathogen preparedness. Pilgrimage-associated respiratory illness is not caused by SARS-CoV-2 alone. Influenza, respiratory syncytial virus, rhinoviruses, endemic coronaviruses, and MERS-CoV all remain relevant to Saudi Arabia and Hajj (8–10, 37–40). Recent progress in RSV and mRNA influenza vaccine development suggests that future preparedness may involve broader respiratory-vaccine strategies, especially for older adults and people with chronic disease (41, 42). The ChAdOx1 MERS vaccine candidate has also generated humoral and cellular immune responses in a phase 1b trial among healthy Middle Eastern adults, reinforcing the importance of maintaining coronavirus vaccine-research capacity in the region (37).

Fifth, public communication should distinguish waning infection protection from durable severe-disease protection. Overstating vaccine capacity to prevent all infections can undermine trust when breakthrough infections occur. A more scientifically accurate message is that vaccination and boosters reduce the probability of severe disease, help protect vulnerable groups, and reduce pressure on healthcare services, even when mild infection remains possible (21–26).

Finally, Saudi Arabia can contribute beyond national policy. Its experience with Hajj, Umrah, MERS-CoV, COVID-19 digital tools, and large-scale public-health operations provides a model for other mass gatherings, including sporting, cultural, humanitarian, and religious events. The next phase of mass-gathering medicine should combine immunology with operational intelligence: vaccine history, immune durability, clinical risk, variant monitoring, and healthcare capacity should be interpreted together rather than separately.

The operational value of immune-durability evidence lies in its translation into preparedness planning. For Hajj, Umrah, and other high-density settings, vaccination policy should be linked to clinical risk, booster timing, heterologous vaccine histories, digital documentation, respiratory-virus surveillance, and public communication. Table 3 presents a preparedness framework that connects immune durability with practical mass-gathering operations.

**Table 3:** Preparedness framework linking immune durability to mass-gathering operations.

Preparedness domain	Actionable interpretation	Examples of implementation
Risk stratification	Clinical vulnerability may matter more than population-level coverage.	Prioritize older adults, pregnant women, immunocompromised individuals, and persons with diabetes, cardiovascular, kidney, lung, or obesity-related risk (9, 19, 20).
Booster timing	Protection against infection may wane faster than protection against severe disease.	Offer risk-based booster advice before predictable exposure periods, particularly for high-risk pilgrims and frontline staff (7, 21, 22, 30).
Heterologous schedules	Pilgrims have diverse vaccine histories and may not fit a single-platform policy.	Allow evidence-based mixed-platform boosting when appropriate, with clear documentation and pharmacovigilance (27–29).
Serological and immune monitoring	Antibody data are useful but incomplete.	Use serology for population trends and targeted studies; add cellular immune endpoints in research cohorts (12, 23–26).
Digital documentation	Certificates should support intelligence, not only access control.	Link vaccination history, date of last dose, risk category, and country of origin while protecting privacy (34).
Respiratory-virus surveillance	COVID-19 preparedness should be integrated with influenza, RSV, MERS-CoV, and other respiratory pathogens.	Combine syndromic surveillance, multiplex testing, genomic sequencing, and hospital outcome monitoring (35–42).
Communication	Public trust requires realistic framing.	Explain that breakthrough infections may occur but vaccination remains important for preventing severe disease and protecting health-system capacity (21–26).

This table translates immune-durability evidence into preparedness actions for Hajj, Umrah, and other mass gatherings. It links risk stratification, booster timing, heterologous schedules, immune monitoring, digital documentation, surveillance, and communication within a layered preparedness framework.

## 8 Limitations

This review has several limitations. First, it is a structured narrative review rather than a systematic review or meta-analysis; therefore, it does not provide pooled estimates of antibody decline, vaccine effectiveness, or booster impact. Second, the included evidence is heterogeneous in study design, vaccine platform, timing of sampling, immune endpoint, laboratory method, and outcome definition. Direct comparison across studies must therefore be cautious.

Third, Saudi-specific evidence remains uneven. Existing Saudi studies provide important data on seroprevalence, humoral responses, vaccine uptake, clinical outcomes, and ICU protection, but longitudinal national data integrating neutralization, memory B cells, CD4+ T cells, CD8+ T cells, variant-specific outcomes, and detailed comorbidity profiles are limited (11–17). Evidence in international pilgrims before and after Hajj or Umrah is even more limited. Fourth, public dashboards are dynamic and depend on testing intensity, reporting criteria, and data-update schedules; the figures cited in this review should be verified against the most recent official sources before policy use.

Fifth, immune correlates of protection continue to evolve as SARS-CoV-2 lineages, booster formulations, and population exposure histories change. Neutralizing antibodies remain valuable correlates of protection against symptomatic infection, but they do not fully capture mucosal immunity, memory B-cell maturation, T-cell protection, or host-level risk. The policy implications presented here should therefore be adapted to real-time epidemiology, vaccine availability, national regulations, WHO guidance, and operational feasibility.

## 9 Future research priorities

Future Saudi research should move from cross-sectional immune description toward longitudinal immune intelligence. Priority studies should measure antibody titres, neutralizing activity, memory B-cell responses, CD4+ T-cell responses, CD8+ T-cell responses, vaccine history, prior infection, comorbidity, age, pregnancy status, immunosuppression, breakthrough infection, hospitalization, and mortality within the same cohorts. Such integration would allow Saudi Arabia to evaluate not only whether immune markers decline, but also which markers best predict clinically meaningful protection.

Hajj and Umrah offer unique opportunities for prospective studies. Pre-travel immune assessment, respiratory-virus testing during pilgrimage, post-travel follow-up, and linkage to clinical outcomes could clarify how vaccine history, time since last exposure, heterologous schedules, and hybrid immunity influence infection and severe disease among pilgrims. These studies should include international participants and should be designed with ethical data governance, multilingual consent processes, and privacy-preserving digital infrastructure.

Research should also evaluate preparedness systems themselves. Digital health certificates, risk stratification algorithms, syndromic surveillance, genomic sequencing, hospital admission triggers, and communication strategies should be assessed as public-health interventions. This would help transform mass-gathering preparedness from a reactive approach into a predictive model that anticipates vulnerability before severe outcomes occur.

## 10 Conclusion

COVID-19 vaccine-induced immunity is durable in some respects and vulnerable in others. Circulating antibodies and neutralizing activity decline over time and can be eroded by immune-escape variants, but memory B-cell and T-cell responses generally provide more sustained protection against severe disease. This distinction is essential for Saudi Arabia, where Hajj and Umrah create predictable periods of intense respiratory-virus exposure among populations with heterogeneous immune histories.

Saudi evidence remains limited but meaningful. National seroprevalence studies, vaccine-rollout data, clinical-effectiveness analyses, and recent serological findings indicate substantial prior exposure, robust post-vaccination responses, and clinically important protection, particularly against severe outcomes and after booster doses. The main policy implication is not that every individual needs the same booster at the same time, but that preparedness should become more precise: risk group, vaccine platform, time since last exposure, prior infection, variant context, and exposure intensity all matter.

For Hajj and Umrah, immune durability should be used as a practical preparedness concept. It can inform booster timing, pilgrim risk stratification, digital vaccination documentation, genomic surveillance, integrated respiratory-virus monitoring, and public communication. By linking immunology with operational mass-gathering experience, Saudi Arabia can strengthen protection for pilgrims while contributing to the wider science of global health security.
